# Supplementary material for: β-hydroxybutyrate accumulates in the rat heart during low-flow ischaemia with implications for functional recovery
Source: eLife. 2021 Sep 7;10:e71270. doi: 10.7554/eLife.71270 (PMC8423437; doi:10.7554/eLife.71270)
Supplement: Supplementary file 1. [file elife-71270-supp1.docx]

**Supplementary File 1: Pre-Ischaemic Cardiac Function for Figure 2 hearts**

| *LVDP (mmHg)* | *Heart Rate (bpm)* | *RPP (mmHg.min)* |
| --- | --- | --- |
| 119 ± 7 | 300 ± 12 | 35500 ± 2500 |
