# Supplementary material for: β-hydroxybutyrate accumulates in the rat heart during low-flow ischaemia with implications for functional recovery
Source: eLife. 2021 Sep 7;10:e71270. doi: 10.7554/eLife.71270 (PMC8423437; doi:10.7554/eLife.71270)
Supplement: Supplementary file 2. [file elife-71270-supp2.docx]

**Supplementary File 2: Pre-ischaemic Contractile Function for Figure 4-figure supplement 2 Functional Recovery**

|  | *LVDP (mmHg)* | *Heart Rate (bpm)* | *RPP (mmHg.bpm)* |
| --- | --- | --- | --- |
| Control Group  (n = 5) | 119.7 ± 5.5 | 271.6 ± 30 | 34300 ± 3000 |
| Oxamate Group  (n = 5) | 130.8 ± 11.2 | 273.5 ± 26 | 34400 ± 540 |
