# Supplementary material for: β-hydroxybutyrate accumulates in the rat heart during low-flow ischaemia with implications for functional recovery
Source: eLife. 2021 Sep 7;10:e71270. doi: 10.7554/eLife.71270 (PMC8423437; doi:10.7554/eLife.71270)
Supplement: Supplementary file 3. [file elife-71270-supp3.docx]

**Supplementary File 3: Pre-ischaemic Contractile Function for Figure 6 Functional Recovery**

|  | *LVDP (mmHg)* | *Heart Rate (bpm)* | *RPP (mmHg.bpm)* |
| --- | --- | --- | --- |
| Control Group  (n = 5) | 120.2 ± 14.1 | 305 ± 15 | 36900 ± 4800 |
| Hymeglusin Group (n = 5) | 107.3 ± 7.5 | 284.9 ± 9.2 | 29700 ± 3300 |
